# Strategies for ultrahigh outputs generation in triboelectric energy harvesting technologies: from fundamentals to devices

**DOI:** 10.1080/14686996.2019.1655663

**Published:** 2019-08-16

**Authors:** Jeong Min Baik, Jin Pyo Lee

**Affiliations:** School of Materials Science and Engineering, Ulsan National Institute of Science and Technology (UNIST), Ulsan, Republic of Korea

**Keywords:** Triboelectric nanogenerator, high electric-outputs, self-powered systems, charged materials, self-charging technology, 102 Porous / Nanoporous / Nanostructured materials, 206 Energy conversion / transport / storage / recovery, 307 Kinetics and energy / mass transport

## Abstract

Since 2012, a triboelectric nanogenerator (TENG) has provided new possibilities to convert tiny and effective mechanical energies into electrical energies by the physical contact of two objects. Over the past few years, with the advancement of materials’ synthesis and device technologies, the TENGs generated a high instantaneous output power of several mW/cm^2^, required to drive various self-powered systems. However, TENGs may suffer from intrinsic and practical limitations such as air breakdown that affect the further increase of the outputs. This article provides a comprehensive review of high-output TENGs from fundamental issues through materials to devices. Finally, we show some strategies for fabricating high-output TENGs.

## Graphical abstract

**Figure:**
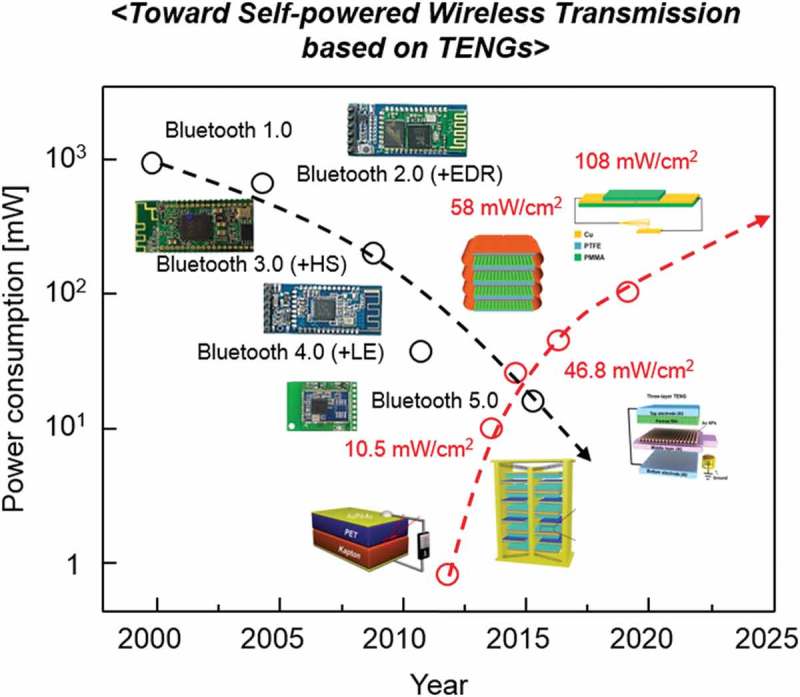

## 1. Introduction

Since the first report of triboelectric nanogenerator (TENG) on 2012, the rapid development of a variety of functional devices and high-performance materials dramatically enhanced the output power, proven to be one of the highly efficient, simple, robust and cost-effective techniques for converting mechanical energies around us to electricity [1–5]. The mechanical energy sources involve a wide variety of classifications for types of energy such as human motion, wind flow, flowing water, vibration and any other mechanical motion. Under surrounding environments of ambient temperature and relative humidity, and under practical input force conditions associated with each energy sources, quite high instantaneous power densities up to several tens of mW/cm^2^ were routinely reported in several papers so far [1,6–11]. The technological advances made the TENGs to ensure continuous and reliable supply of power for many devices such as wearable devices, sensors, smartphones and medical devices, giving the realization of various self-powered systems [8,12–17].

**Figure 1.**
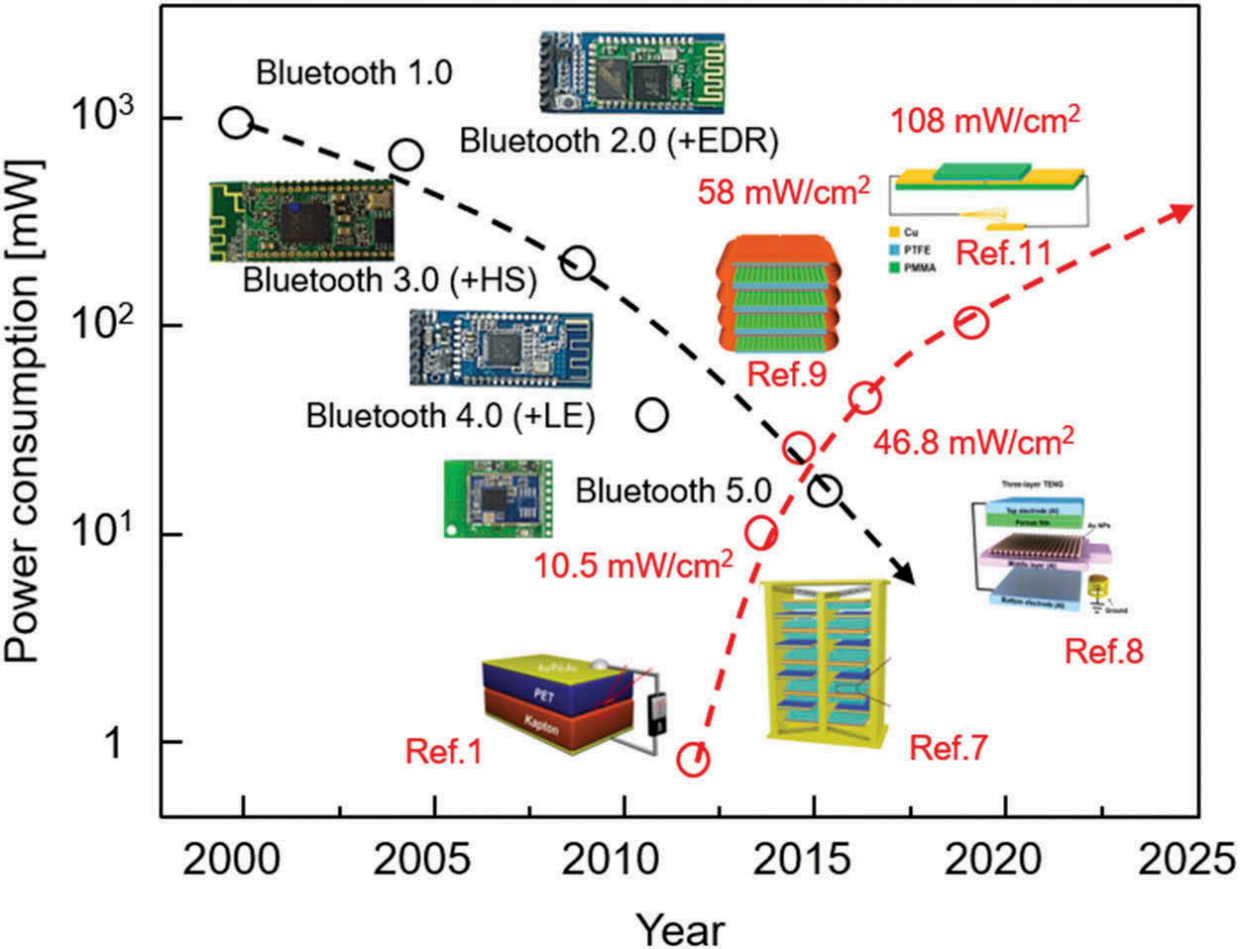
Power consumption for Bluetooth and instantaneous output powers of various TENGs.

Among many potential applications in the near future, the self-powered wireless communication technologies may be quite attractive because of the broad applicability such as sensor network system, security systems, intelligent transportation systems and patient monitoring and telemedicine [18,19]. So far, the technologies have transformed the means of information exchange or sharing, communication and transactions, leading to the new digital economy. They are also deeply related with the realization of The Fourth Industrial Revolution, stimulated by numerous internet of things (IOT) sensors and wireless transmission of data or information. Thus, the global market in the wireless data communication market was reported to be valued at 794.6 million USD in 2018, expected to reach 1867.8 million USD by 2023 [20]. Introducing a new concept of self-powered technologies will allow many devices to be free from any power cables or battery-changing task, which will further increase the market, more than as expected. Figure 1 shows the power consumption of the Bluetooth, compared with the instantaneous output power densities of various TENGs developed so far. It is obviously seen that the Bluetooth energy has continuously decreased, while the output power of the TENGs has increased up to several mW/cm^2^ [1,7–9,11]. This means that the output power may become comparable to the Bluetooth energy in the near future. However, for the successful implementation of TENGs in practical applications, it is essential to store the sufficient output power in a capacitor or a battery, to operate the devices. Although there was some progress in increasing the charging efficiency via the optimized circuit design, the efficiency was still so low [21,22].

In general, many strategies to increase the output powers of TENGs are based on the enhancement of the charge transfer occurring between two contacted surfaces to increase the transferred charge density because it determines the electric potential between two electrodes. According to the triboelectric series, many combinations associated with various polymers have been applied to various TENGs to show quite high charge densities up to several hundred μC/m^2^ [6,8,10,23,24]. Some advances associated with the contacted materials involved electronic and structural modifications, such as a large work-function difference, high porosity, large dielectric constant and large surface roughness [17,25–32]. The maximum charge density of these TENGs in practical environments, however, requires additional processes, such as artificial charge injection and poling process under high electric field [6,10,33]. Recently, several modifications to amplify the current flowing through the external circuit have been successfully demonstrated [34,35]. Metal-dielectric-metal structures or metal-metal contacts have been introduced and have been proven to increase the charge density of the TENG by several times [8,36]. Here, we review various strategies for ultrahigh outputs generation in triboelectric energy harvesting technologies from the charge transfer mechanism to the fabrication of the devices.

## 2. Contact-electrification effect

Contact electrification (CE) is the charging of the surfaces due to the charge transfer occurring during the physical contact between two materials. The CE has been studied for a long time (~2600 years) ago since Thales of Miletus showed that rubbing amber against wool could make electrostatic charges [37]. Actually, the CE routinely occurs around us and common sense tells us that it is strengthened in a dry environment. Despite of its long history and practical importance, there have still been many debates about the origin of the CE and strategies to control the CE were not investigated systematically yet.

In general, the charge transfer mechanism has been explained via the transfer of ions and electrons according to types of the contacted materials [38–44]. In insulator-insulator systems, the ion transfer was proposed to explain the electrification, evident in CE related with the polymers containing mobile ions. The electron transfer mechanism has been mainly applied to the metal-polymer systems although the ion transfer has been also useful in a few light contacts. In this mechanism, physical concepts such as effective work function and density of states have been utilized in equilibrium model, in which it was important to lower the charge-neutrality level of the surface states to increase the transferred charge density. A local model predicted that the charge exchange could be determined by the localized structure of the polymers, that is, the number and the position of insulator states. If the states were tuned on the basis of the energies and positions by introducing various functional groups, electron-transfer and charge-retention characteristics of the polymers would change accordingly. The molecular ion state model also supported the charge carrier transfer between polymers, leading to the formation of anion states and accepter states. However, the charges carrying states in polymers were similar to the ions in solution.

## 3. Effective charged materials

**Figure 2.**
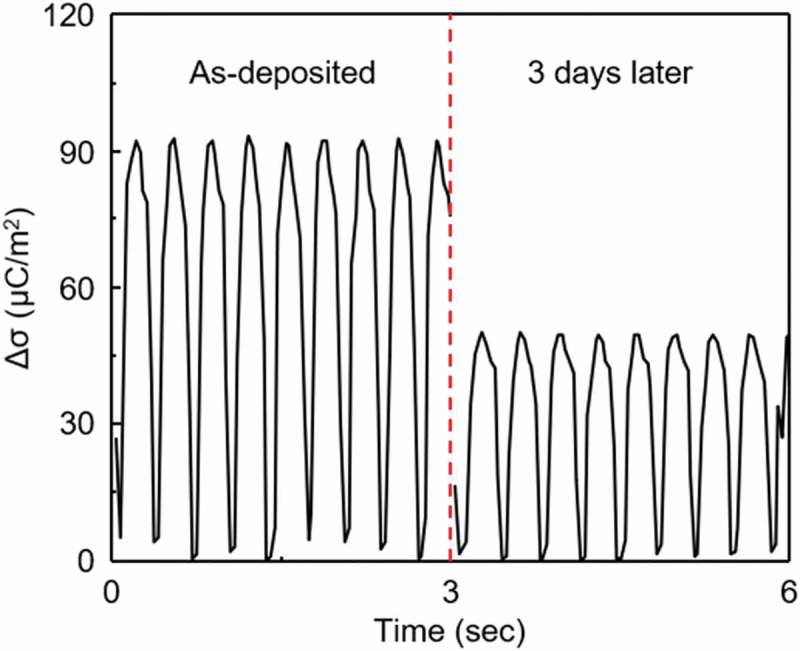
The charge densities of the TENGs composed of Al and PTFE layer. The Al was deposited by e-beam evaporation and the charge densities were measured after 3 days in air.

According to the suggested CE mechanisms and triboelectric series, most researchers have tried to find the best combinations of metals and polymers for maximizing the charge density. As positively charged materials, aluminum (Al) as a form of foil and film, was widely used because of the excellent electron donor characteristics although there have been promising candidates consisting of gold (Au), copper (Cu), etc. [6,11,12,43,45,46]. Actually, the strategy for the positively charged material may be simple. According to the electron transfer mechanism, the materials with the lowest work function will be the best candidates, such as Al, zinc (Zn), etc. However, the surface of such metals may be easily oxidized and reacted, thus, a thin oxide layer was always formed [40,47]. A TENG composed of Al and polytetrafluoroethylene (PTFE) layer was fabricated, in which the Al was deposited by e-beam evaporation onto indium tin oxide substrate. The charge density was measured to be about 90 μC/m^2^ in a nitrogen atmosphere, but after 3 days in air, it decreased to 50 μC/m^2^, as shown in Figure 2. Other issue to be considered is the stretchability of the metals. The metals are quite rigid and once bent or wrinkled, they are not easily restored. Recently, Kim et al. synthesized a stretchable conductive layer composed of Ag nanowires-polydimethylsiloxane (PDMS) composite, onto which 4-dimethylaminopyridine (DMAP)-capped Au nanoparticles were coated to reduce the work function of the surface layer [48]. The stretchable layer played an important role in enhancing the contact uniformity, increasing the charge density on the surface of the polyimide (PI) layer. The surface of Au was modified with thiol molecules by using self-assembled monolayer (SAM), lowering the electronegativity at the surface, and the output power of the TENG fabricated with fluorinated ethylene propylene (FEP) was enhanced by ~4 times [49].

Negatively charged materials can be much more complex than positively charged materials. It is well known that the charge density is influenced by the surface chemical potential difference between the two contacted materials, according to the following general equation [40],

$$\sigma =\frac{\left[\left(W-E_{0}\right)e\right]\left(1+t\;/\;\varepsilon z\right)}{t\;/\;\varepsilon \varepsilon_{0}+\left(1/\bar{N_{s}\left(E\right)}e^{2}\right)\left(1+t\;/\;\varepsilon z\right)}$$

where $N_{s}\left(E\right)$ is the density of surface states (m^−2^∙eV^−1^), W is the work function of the metal, $E_{0}$ is the charge-neutrality level of the surface states, e, t, ε, ε_0_ and z are the charge of an electron, distance of space, relative permittivity of dielectric, vacuum permittivity of free space and thickness of dielectric film. The charge-neutrality level is the energy level at which the interface at the metal-polymer system is electrically neutral; it is determined by considering all molecular states as well as the highest occupied molecular orbital (HOMO) and lowest unoccupied molecular orbital (LUMO) resulting from interfacial interactions. In some polymers, the level is known to be located close to the LUMO as the density of states is higher around the HOMO [38]. The above equation indicates that as the position of the charge-neutrality level becomes lower, the surface charge density increases accordingly.

**Figure 3.**
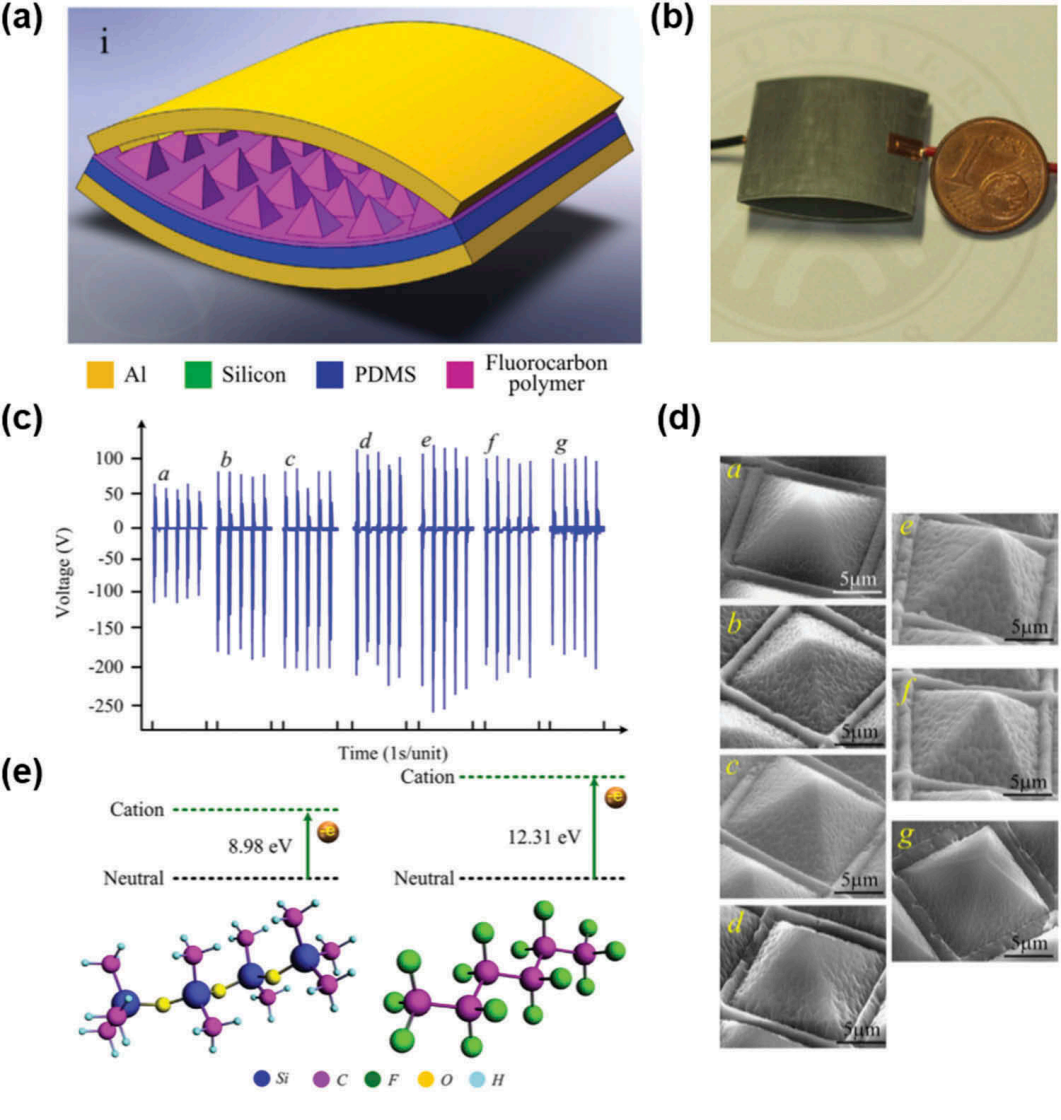
(a) Schematic view of 3D structure and (b) photo of the high-performance triboelectric nanogenerator. (c) Output voltage waveform of the triboelectric nanogenerator with different surface chemical modifications (i.e., plasma treatment cycle) under the external force with a frequency of 5 Hz. (a-0 cycle, b-1 cycle, c-2 cycles, d-4 cycles, e-8 cycles, f-10 cycles, g-20 cycles). (d) SEM images. (e) The theoretical calculation results of vertical ionization energy of model complexes of PDMS and fluorocarbon layer deposited by the C_4_F_8_ plasma treatment. Copyright 2014 Elsevier. Reproduced with permission from [50].

At the beginning of the research, most researchers focused on the electronegativity of polymers because it was deeply related with charge-neutrality level. Electronegativity refers to the ability of an atom to attract the electrons shared by atoms in a covalent bond. The higher the value of the electronegativity, the more strongly the atom attracts the electrons. Fluoro group (-F) has the highest tendency to attract electrons, thus, various polymers with the fluoro group such as PTFE, FEP and polyvinylidene fluoride (PVDF) were tested and quite high instantaneous outputs were reported in various-type TENGs [45,49,50]. Recently, the surface of PDMS in an arch-shaped TENG was modified via fluorocarbon (C_4_F_8_) plasma treatment (Figure 3(a,b)), the TENG output peak voltage increased sharply from 124 V to 265 V after eight-cycle plasma treatment (Figure 3(c)). The energy volume density of TENG was enhanced by approximately 278%, compared with the TENG fabricated with pristine PDMS layer. The enhancement of the outputs was attributed to the increase of the ionization energy by fluorination and the formation of PDMS micro/nano hierarchical structures by the treatment (Figure 3(d,e)).

To increase the surface roughness has been commonly used to increase the contact area between two materials. Various patterns with various features, such as lines, cubes, pyramids and nanowires on the surface are made by a simple patterning process and a reactive ion etching (RIE) process, respectively [3,45,51]. PDMS was widely used as an effective dielectric because it was quite viscoelastic due to the flexible polymer chain by their siloxane linkages, by a simple curing process. Thus, various patterns were fabricated by using conventional patterning processes. However, there have been still debates about the relationship between the surface roughness and the contact area because the polymers were generally soft and compressible, compared with the metals. Recently, Dudem et al. demonstrated that the contact force, defined as a product of effective/normal stress and contact area, contributed to the increase of the surface charge density by investigating the relationship between the period of nanopillar array on PDMS surface and the contact force, and the outputs of the TENGs [52].

**Figure 4.**
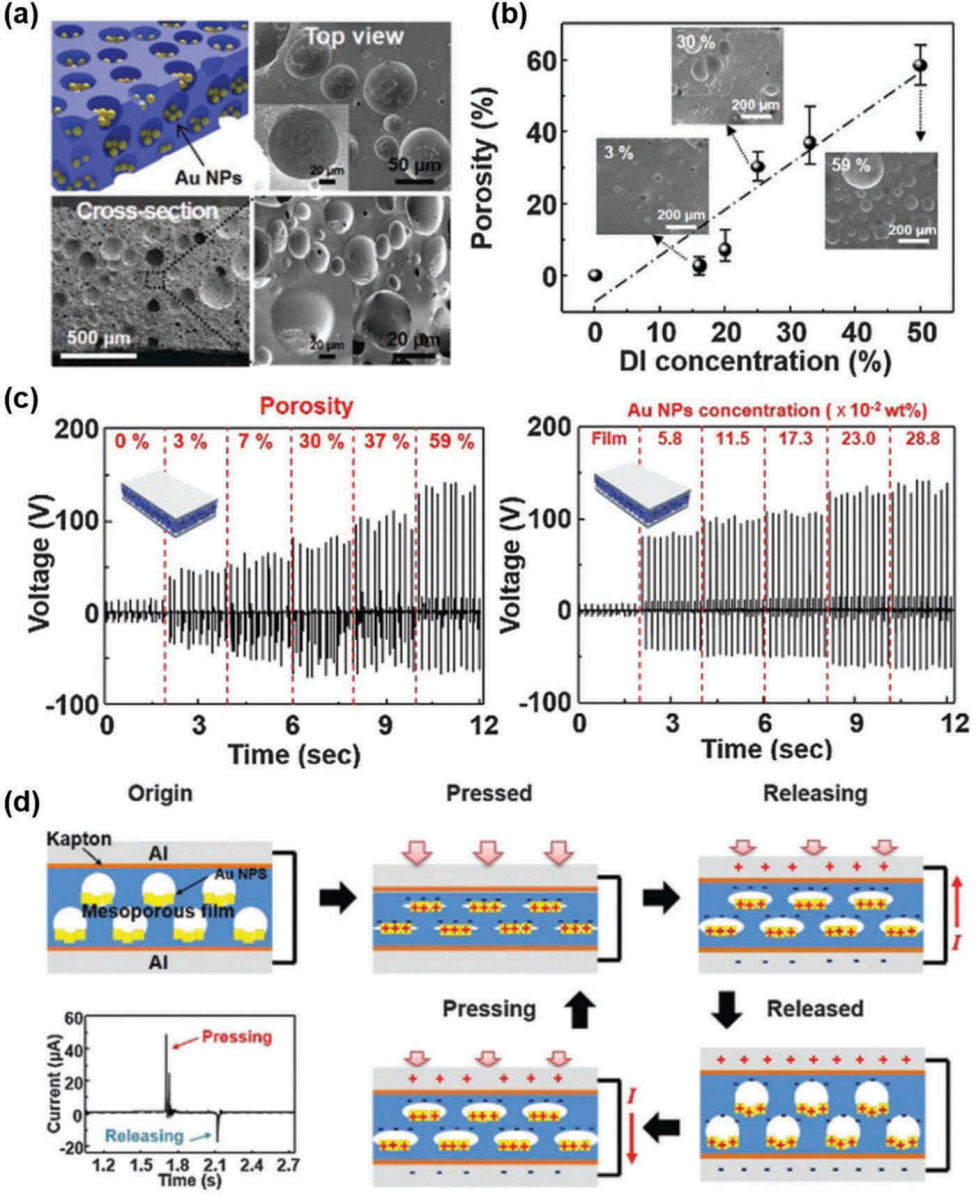
(a) Top and cross-sectional SEM images of a mesoporous PDMS film with Au NPs (0.28 wt%). (b) Porosity changes as a function of the DI water concentration ranging from 0% to 50%. The insets show SEM images of mesoporous films with various porosities. (c) The output voltages generated by the AMTENG as a function of the porosity ranging from 0% to 59% at a fixed Au content (0.28 wt%) and the Au NP concentration ranging from 5.8 to 28.8 × 10^−2^ wt%. (d) The charge generation mechanism of the AMTENG under an external force and short-circuit conditions. Copyright 2015 Royal Society of Chemistry. Reproduced with permission from [27].

The mechanical properties of the polymers were further investigated by introducing micropores to the polymers. According to the following equation,

$$I=\frac{\partial Q}{\partial t}=V\frac{\partial C}{\partial t}+C\frac{\partial V}{\partial t}$$

the output current of the TENG can be determined by the capacitance change and the capacitance of the polymer. However, from materials aspect, the two factors are in negative correlation when determine the power, that is, if the capacitance change increases, the film becomes compressible and the capacitance decreases due to the presence of air in the pores. Various mesoporous films such as PDMS, PTFE and PVDF were achieved by the use as sacrificial materials such as water and ZnO [27,53]. The electrical outputs of the TENGs fabricated with the mesoporous films were enhanced by several times due to the increase of the contact area and the film displacement. Interestingly, the TENGs showed stable output performance less sensitive to humidity. Chun et al. also demonstrated a TENG without air gap by impregnating Au nanoparticles into the pores. A mesoporous film was fabricated after removal of DI water and Au NPs inside the pores, and the Au nanoparticles were naturally positioned on the bottom side of pores (Figure 4(a)). The porosity increased to approximately 59% as the amount of water increased to 50% (Figure 4(b)). It was also possible to fabricate a large-area mesoporous PDMS thin film (30 cm × 30 cm) and the film was seen to be quite flexible by the stress–strain curve measurement. The output voltages significantly increased with the porosity and the Au nanoparticles concentration, as shown in Figure 4(c). The aligned dipoles produced due to the charges created by the contact between Au and PDMS inside the pores explained the increase of the electrical potential (Figure 4(d)).

As for the capacitance change, the capacitance, that is, the dielectric constant value should be increased. In general, polymer composites composed of high dielectric materials were widely used as effective dielectrics to enhance the output power of the TENGs [10,54–56] Ferroelectric nanoparticles such as BaTiO_3_ increased the surface charge potential, enhancing the charge transfer behavior. The charge-trapping capability was also enhanced via the increase of the dielectric constant, thus, providing larger output current. However, due to the different mechanical properties of the organic and inorganic materials, the materials can be unstable during the operation for a long time. Lee et al. reported a robust nanogenerator based on poly(tert-butyl acrylate) (PtBA)–grafted PVDF copolymers via dielectric constant control through an atom transfer radical polymerization technique [30]. The grafting of PtBA onto the PVDF backbone increased the dielectric constant from 8.6 to 16.5 at the grafting ratio of 18%, enhancing the instantaneous output power by 20 times. Here, a linear relationship between the dielectric constants and output currents of the TENG was clearly proved by measuring saturated times and currents of PVDF-based TENGs with grafting ratios.

**Figure 5.**
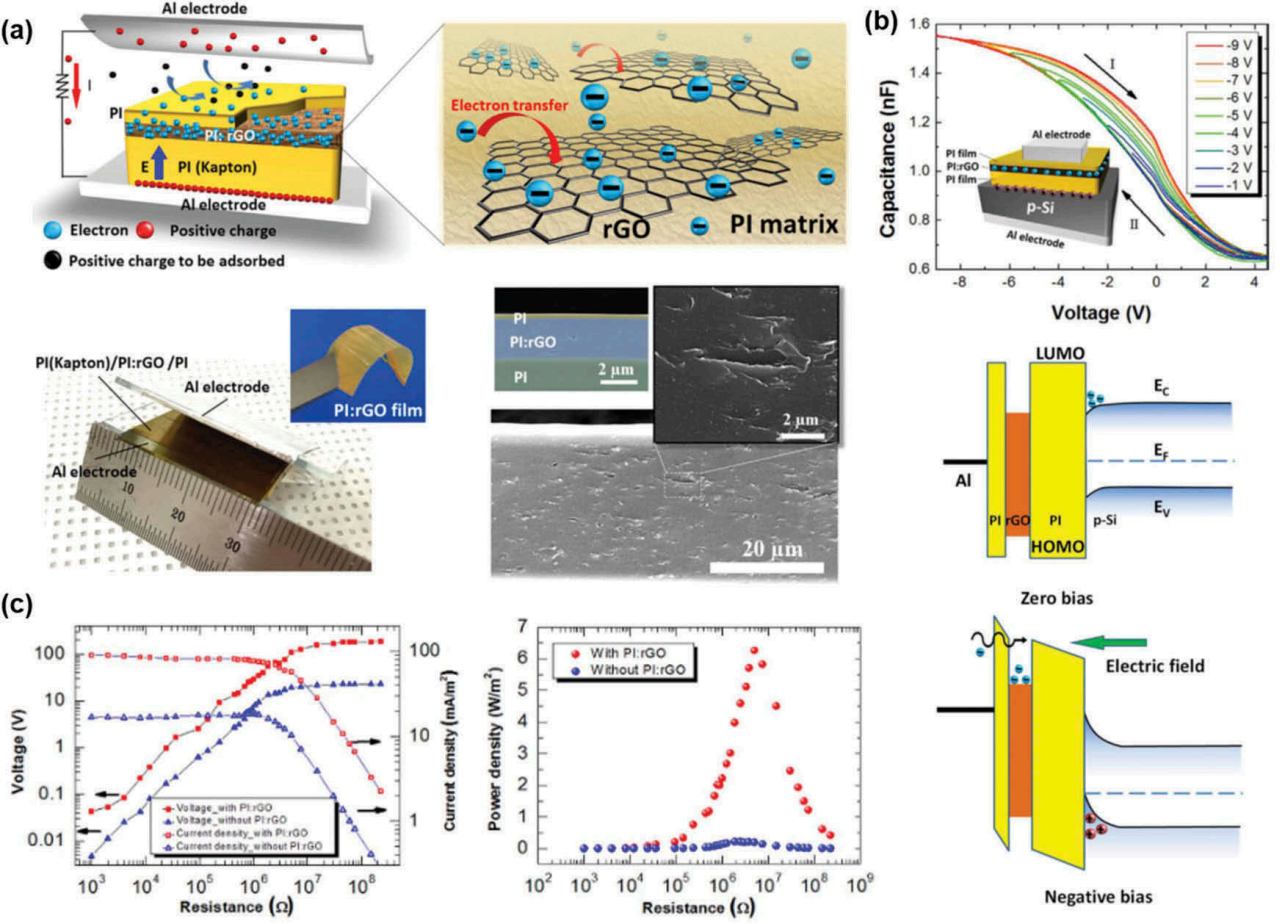
(a) Illustration of the vertical contact-separation mode TENG with a PI:rGO film. The right panel shows a schematic diagram of electron transfer from the PI layer to the rGO sheets. Optical image of the vertical contact-separation mode TENG. The inset presents an optical image of the separated PI:GO layer. Scanning electron microscopy (SEM) images of the PI:rGO layer. The inset presents an SEM image of the PI (Kapton)/PI:rGO/PI stacked layer. (b) C-V curves of the Al/p-Si/PI/PI:rGO/PI/Al device. The inset presents a schematic of the device used for the C-V measurements. Schematic diagram of the energy bands for the floating-gate metal-insulator-semiconductor (MIS) device under zero bias (up) and under negative bias (down). (c) Dependence of the output voltage and the current density on the external loading resistance (left). Dependence of the output power density on the external loading resistance (right). Copyright 2017 Elsevier. Reproduced with permission from [59].

So far, various single-layered films were reviewed as effective dielectrics in TENGs. Recently, multi-layered films have been fabricated by introducing electron accepting layers such as polystyrene (PS), MoS2, reduced graphene oxide (rGO) and TiO_x_ [57–60]. Cui et al. suggested a new concept that more charges would be trapped inside the layers via the abundant trap levels of electrons that the PS had. The insertion of the PS layer between PVDF and Al electrode significantly increased the charge density by 7 times, compared with pristine PVDF film. Wu et al. tried to explain the increase of the charge density via the energy band diagram of PI/rGO/PI (Figure 5(a)). During the physical contact between the top PI layer and the Al electrode, electrons were transferred to PI and as the external forces are released, an electric field directed from the bottom substrate to the top surface was generated by the positive charges driven in the bottom electrode. Thus, the transferred electrons will be captured into the rGO layer along with quantum confinement effects, supported by the C-V measurements by applying voltage sweeps from −1 to −9 V (Figure 5(b)). This has increased the TENG output to 6.3 W/m^2^ (Figure 5(c)). Lee et al. reported that the electric outputs of the TENGs were enhanced by several tens percent when the two-layered dielectric film composed of the PVDF and PI-BTO was used [22]. The enhanced charge density was interpreted in terms of the two electrostatic forces, acting between the top electrode and the dielectric, and the dielectric and the bottom electrode. To maximize the charge density between the electrodes, the two forces needed to be balanced.

The charge density was also increased by ions injection as well as the contact electrification. The charge injection in TENG by using corona discharging was studied because the maximum charge density would be limited by the air breakdown, considered a straightforward way to further increase the charge density [6,58]. In general, the dielectric strengths of the polymers used as dielectrics are larger than that of the air, indicating that the charge density can be further increased by the ion-injection process. Wang et al. reported that the negative ions such as CO_3_^−^, NO_3_^−^, NO_2_^−^, O_3_^−^ and O_2_^−^ were injected onto the surface of FEP, making it negatively charged. The charge density was increased by approximately 5 times, to 240 μC/m^2^ after a few times of injection. The process was applied to various dielectrics such as PTFE/PA6(polyamide-6), PTFE and a parylene C/polyimide/SiO_2_. However, the charge densities of the TENGs fabricated with a single-layer were still not increased, although a little increase was observed in the multilayered film.

## 4. Upcoming breakthrough technologies toward high-output generation

Very high electric potentials in the range of several hundred to several thousand voltages were commonly generated between the two separated materials with the opposite triboelectric charges. Wang et al. reported that a high voltage of 1.5 × 10^5^ V would be induced with a charge density of 300 μC/m^2^ [46]. When the dielectric was stressed by such high voltage (that is, high electric field), the air and the dielectric can begin to break down, becoming partially conductive. Table 1 shows the dielectric constants and dielectric strengths of various materials at room temperature [61–63]. The dielectric strength is the maximum electric field that can exist in a dielectric without electric breakdown. The electric fields are in the range of several tens of 10^6^ V/cm, not significantly dependent on the dielectric constant. Thus, the breakdown mechanism may limit the maximum retainable charge density which can be generated in TENGs.

**Table 1.** Dielectric constants and dielectric strengths of various materials at room temperature.

Material	Dielectric constant (k)	Dielectric Strength (MV/m)
Air (dry)	1.00059	3
Nylon	3.4	14
Polystyrene	2.56	24
Polyvinyl chloride	3.4	40
Polytetrafluoroethylene	2.1	60–173
Polyvinylidenefluoride [61]	8.4	64.2
Polyimide [62]	3.4	2–3.5
Polydimethylsiloxane [63]	2.3–2.8	65

To overcome the limitation of the materials, a new type of TENGs was suggested, based on metal-metal contact for current amplification [8,36]. In the structure, an Al layer was added underneath the positive-charged layer (i.e. Al) and the direct electrical connection of the positive-charged layer to the earth (ground) induced the efficient charge separation in the layer, based on Volta’s electrophorus. This provided substantially larger electric potential at even low-frequency regime, producing an enhanced instantaneous output current of 1.22 mA. The shape of the single current peak was seen to be totally different from those in two-layered TENGs. Chung et al. also demonstrated a new TENG generation mechanism involving the Leyden jar effect, where a capacitor was combined with the TENG. The TENG was composed of a capacitor, a TENG unit and a cylinder containing the TENG. In the capacitor, the charges that the TENG generated were temporarily stored and by the metal-metal contact, they were released to the electrode, which produced a large peak current. Interestingly, the internal impedance of the TENG was decreased to 100 kΩ by the contact.

**Figure 6.**
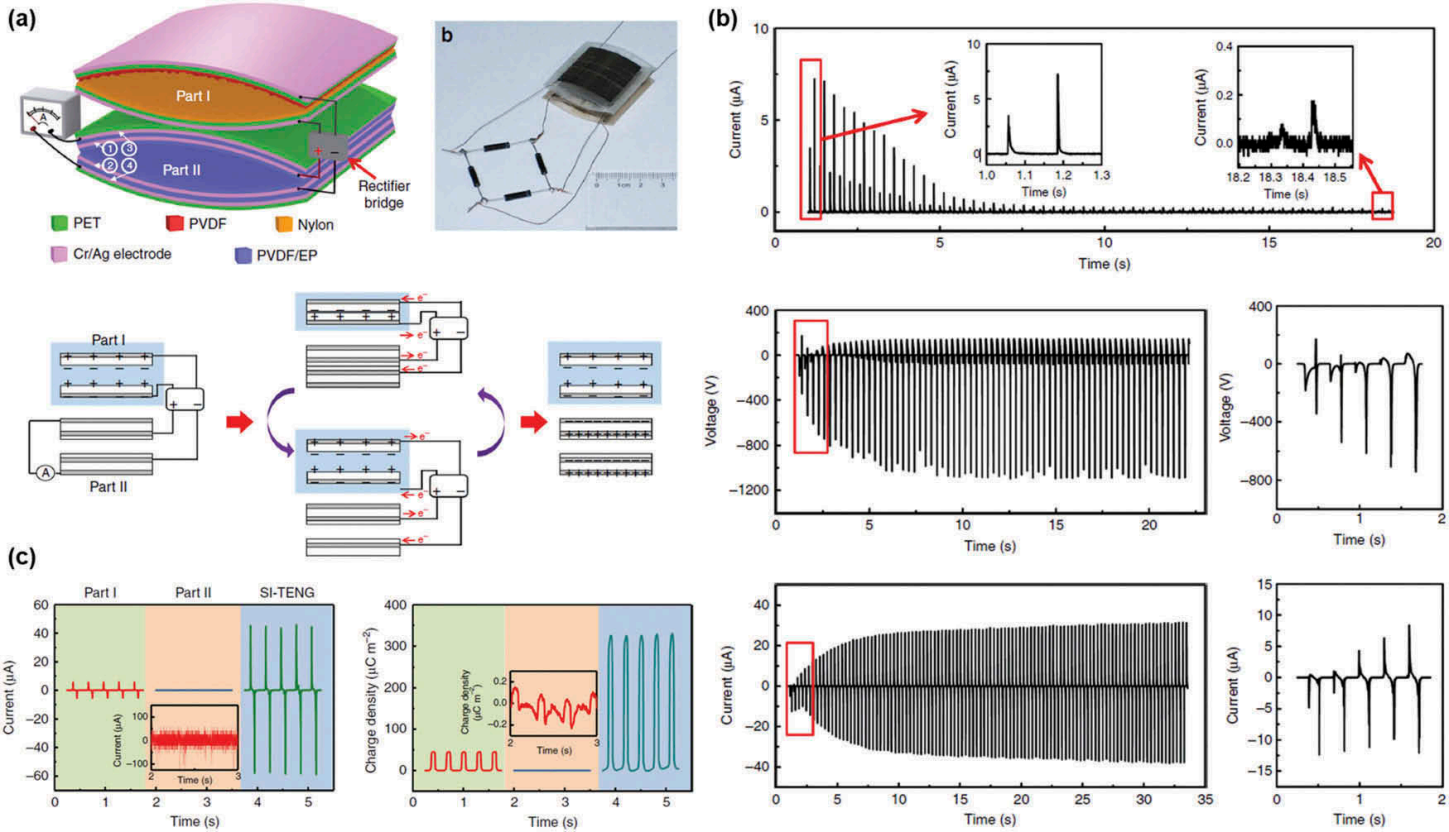
(a) Schematic diagram of the self-improving triboelectric composed of PET, PVDF and PVDF/epoxy resin (EP). Optical image of a SI-TENG. Charge accumulating process of the SI-TENG and corresponding charge transfer direction in the circuit. The friction layers in part I are replaced by positive and negative charges, and the PET film in part II are not shown in the image. (b) The current measured at the positive pole of the rectifier bridge shows the charge generated by the voltage source filled into the plane-parallel capacitor structure (PPCS). Output voltage and current of the self-improving triboelectric nanogenerator increase with charge accumulation in the PPCS. (c) The output current and the transferred charge density, respectively, of part I (left lines), part II (middle lines) and the self-improving triboelectric nanogenerator (SI-TENG). Copyright 2018 Springer Nature. Reproduced with permission from [34].

Recently, self-charging technologies in TENGs for further increase of the charge density were demonstrated by using a network of capacitors and diodes [34,35,64]. Cheng et al. reported a self-improving TENG composed of two parts, that is, a TENG in vertical contact-separation mode which generates charges and a plane-parallel capacitor to store the charges generated in TENG (Figure 6(a)). The two parts were stacked vertically, connected with a rectifier bridge to convert the alternating current (AC) voltage to the direct current (DC) voltage in one direction. Figure 6(b) shows the current flowing from the rectifier bridge into two parallel capacitors, which increased the voltage between two electrodes of the capacitors. By this process, the charge density was increased from 45 μC/m^2^ to 325 μC/m^2^ (Figure 6(c)). Furthermore, ions injection method significantly increased the charge density to 490 μC/m^2^. Liu et al. further increased the charge density to 1250 μC/m^2^ by utilizing voltage-multiplying circuits consisting of Zener diodes and capacitors. In this paper, the two parts were placed in parallel. The technologies are expected to be a promising strategy for high-output TENGs toward practical applications; however, additional space is required for the circuits and one additional device. Accurate control in the operation of the two parts may be also needed.

## 4. Conclusions

We have summarized the recent progress in the development of high-output TENGs from fundamentals to devices. Since the discovery of the TENG on 2012, various TENG devices including the materials’ synthesis technologies generated quite high instantaneous power densities up to several tens of mW/cm^2^ under practical input force conditions associated with various mechanical energy sources. Because of the rapid development during a few years, various potential applications such as portable power source and self-powered sensors successful demonstrated, giving the possibility of the commercialization, especially, related with self-powered wireless transmission technologies. The charge density of the TENGs was continuously increased, by introducing ions injection and poling process under high electric field to multi-layered film or composites-type film. Further increase of the charge density may be limited by air breakdown, meaning that designing concepts for new devices are needed, such as metal–metal contacts and self-charging technologies. We believe that this review can be useful and helpful for designing energy harvesters which are possible to provide sufficient energy with portable electronic devices.
